# Effects of Dietary Lanthanum Chloride on Growth Performance, Hematology and Serum Biochemistry of Juvenile *Clarias gariepinus* Catfish Fed Diets Amended with Mixtures of Aflatoxin B1 and Fumonisin B1

**DOI:** 10.3390/toxins14080553

**Published:** 2022-08-14

**Authors:** Bolade Thomas Adeyemo, Ndidi Gloria Enefe, Tanimomo Babatunde Kayode, Augustina Ezekwesili, Olatunde Hamza Olabode, Audu Zakariya, Gbenga Michael Oladele, Samson Eneojo Abalaka, Wesley Daniel Nafarnda, Clement Barikuma Innocent Alawa

**Affiliations:** 1 Department of Animal Health and Production, Faculty of Veterinary Medicine, University of Abuja, Gwagwalada 902001, Nigeria; 2 Department of Physiology and Biochemistry, Faculty of Veterinary Medicine, University of Abuja, Gwagwalada 902001, Nigeria; 3 Department of Veterinary Microbiology, Faculty of Veterinary Medicine, University of Abuja, Gwagwalada 902001, Nigeria; 4 Department of Veterinary Pharmacology, Faculty of Veterinary Medicine, University of Abuja, Gwagwalada 902001, Nigeria; 5 Department of Veterinary Pathology, Faculty of Veterinary Medicine, University of Abuja, Gwagwalada 902001, Nigeria; 6 Department of Veterinary Public Health and Preventive Medicine, Faculty of Veterinary Medicine, University of Abuja, Gwagwalada 902001, Nigeria

**Keywords:** aflatoxin B1, fumonisin B1, lanthanum chloride, *Clarias gariepinus*, hematology, serum biochemistry

## Abstract

This study aimed to determine the effects of dietary lanthanum chloride on the growth and health performance of juvenile *Clarias gariepinus* when fed diets experimentally contaminated with mixtures of aflatoxin B1 and fumonisin B1. A control diet, (mycotoxin free, diet A), mycotoxin contaminated (diet B), and two mycotoxin-contaminated diets amended with lanthanum chloride (200 mg/kg, diet C; and 400 mg/kg, diet D), were fed to 450 fish divided equally into five groups (each with three replicates) for 56 days. The fish were randomly sampled at the time points: day 7, 28 and day 56 for the zootechnical, hematological and serum biochemical evaluations. The fish fed the diets amended with lanthanum chloride exhibited significantly (*p* < 0.05) better performance indices compared with the fish fed only the mycotoxin-contaminated diet. Lanthanum chloride elicited significant (*p* < 0.05) increases in erythrocytes and leucocytes count and significant (*p* < 0.05) reduction in serum transaminase, alkaline phosphatase, lactate dehydrogenase activities, urea and uric acid concentrations in the fish fed the diets contaminated with mixtures of aflatoxin B1 and fumonisin B1. The study indicates that juvenile *Clarias gariepinus* may be beneficially cultured with mycotoxin-contaminated grains amended with 200 to 400 mg/kg lanthanum chloride.

## 1. Introduction

Aquaculture production represents a significant source of animal protein to millions of people worldwide. According to the Food and Agriculture Organization of the United Nations [1], the total world aquaculture production has risen to 51.4 million tons by volume and $60.0 billion by value. The increase in the production of cultured fish has also led to a significant decrease in the landings of several capture fisheries, a direct consequence of the use of fish as the source of animal proteins in aquafeeds [2,3,4]. To mitigate this negative trend, plant-based proteins are increasingly being used as sustainable alternatives to fish-meal-based proteins in aquafeeds [5].

In Africa, the quality of the plant products used in fish feed formulations is said to be a limiting factor in the progressive increase in aquaculture productivity; in addition, these feed ingredients are, very often, ideal substrates for the growth of fungi, such as *Aspergillus* spp. and *Fusarium* spp. [6,7]. These fungi, under favorable conditions (that is, those generally present in the tropical regions of the world), may result in the synthesis of mycotoxins, such as aflatoxin B1 and fumonisin B1 [4].

The aflatoxins are a group of mycotoxins produced by the blue-green molds, *Aspergillus flavus* and *Aspergillus parasiticus* [8,9]. These molds are common contaminants in the feed ingredients of agricultural origin (such as cotton seed, ground nut, maize, wheat, soya bean and the respective by-products from the agricultural processing of these commodities). The aflatoxins have also been reported in fish meal. Four major aflatoxins (AFB_1_, AFB_2_, AFG_1_ and AFG_2_) have been reported to be direct contaminants of feed ingredients and formulated agricultural and aquacultural feeds [10,11]. Of the aflatoxins, AFB_1_ is reported to be the most prevalent, most potent and the most carcinogenic [12,13,14], and has been classified as a group 1 carcinogen by the International Agency for Research on Cancer [15].

Aflatoxicosis, a disease state caused by the effects of aflatoxins, has been noted to be common in aquaculture [16]. According to [17], *Oncorhynchus mykiss* fed cotton seed meals contaminated with aflatoxins developed liver tumors and exhibited a mortality pattern of up to 85%. Other information available on the effects of AFB_1_ in the cultivable species of fin and non-fin fishes, i.e., *Oncorhynchus mykiss* [18]; *Ictalurus punctatus* [19,20]; *Oreochromis niloticus* [21]; *Labeo rohita* [22] and *Penaeus monodons* [23], suggests that fish exhibit a wide plasticity in the susceptibility to AFB_1_, and that cold water species are more sensitive when compared to warm water fishes [18,20]. These species-specific differences in sensitivity to AFB_1_ have been attributed to the differences in the metabolism of aflatoxin B1 in the liver and the affinity of AFB_1_-derived metabolites to hepatic macromolecules [14,24].

*Fusariumverticillioides,* the mold primarily associated with the production of the fumonisins, is prevalent in hot–humid regions of the world, and its occurrence has been related to the presence of invading insects [24,25]. Though maize is most frequently contaminated by fumonisins, these mycotoxins have been found at high concentrations in wheat, asparagus, tea and cowpea [26]. According to [27], it is very difficult to obtain uncontaminated maize, even if the contamination level is not significant. In most investigations, FB_1_ is the most prevalent toxin, with a co-occurrence of FB_2_ and FB_3_ [25]. Several countries in Africa, North and South America, Asia and Europe have reported FB_1_ in cereals at levels from 0.02 to 25.9 ng/kg, and FB_2_ at levels from 0.05 to 11.3 ng/kg [26,28]. FB_1_ is stable in acetonitrile-water (1:1), at food-processing temperatures and light, but unstable in methanol [26].

Feed additives are used world-wide for many different reasons. Some help to cover the need for essential nutrients, and others help to increase animal performance, feed intake and thereby optimize feed utilization. The rare earth elements (REE), composed of about 15 elements with atomic numbers ranging from 57 (lanthanum) to 71 (lutetium), are a promising set of feed additives in animal production [29,30]. The REE are reported as being used as performance enhancers in animal production, without affecting the quality of the final produce [31,32].

The growth-promoting effects of REE are reported to be based on the type and concentration of the REE applied [33,34]. It is reported that the application of REE additives, with concentrations ranging from 100 to 200 mg/kg, in the diets of 40–50 days old piglets, significantly improved the daily body weight gain [35]. It is reported that diets supplemented with mixtures of lanthanum chloride at 100, 200 and 300 mg/kg per diet improved the activities of proteinase, lipase and amylase in the liver and pancreas of the adults and fry of carp (*Cyprinus carpio*) [36]. Furthermore, dietary lanthanum at 75 mg/kg has been reported to result in a 2–5% increase in body weight and a 7% increase in the feed conversion ratio of piglets [31].

*Clarias gariepinus,* also called the African sharp tooth fish, is widely farmed in the West African sub-region; this fish is cultured based on the aquafeeds produced from maize and soybean cake [37,38], resulting in the risk of inadvertent dietary AFB_1_ and/or FB_1_ exposures. In a previous study, we observed a marked decrease in weight gain following dietary exposures to mixtures of AFB_1_ and FB_1_, and reported the tolerable limits for dietary exposures to mixtures of AFB_1_ and FB_1_ in juvenile *Clarias gariepinus* to be 17.6 μg AFB_1_/kg and 24.5 mg FB_1_/kg [39].

The addition of rare earth metals to animal diets as growth promoters is considered to be a promising alternative to the use of antibiotics and other chemicals [29,40,41]. There are reports on the potential for the use of lanthanum chloride (LC), either as immunostimulants and/or growth-promoters in agriculture, as well as in aquaculture [31,42]. The present study was set up to determine the effects of lanthanum chloride on the growth performance, hematology and serum chemistry of the juvenile *Clarias gariepinus,* when fed diets contaminated with mixtures of aflatoxin B1 and fumonisin B1.

## 2. Results

The nutrient composition, proximate composition and the mycotoxin analysis of the experimental diets are shown in Table 1. It shows that the concentrations of AFB_1_ and FB_1_ in the produced diets are significantly higher than the concentrations of the purified mycotoxins added to the experimental diets at the time of the diet formulation and feed production. Hence, the concentrations of the mycotoxins in the diets would, hereafter, be appropriately quantified as follows: Diet A, the control diet, 2.0 µg AFB_1_; 3.0 mg FB_1_/kg diet; Diets B, C and D, 19.7 µg AFB_1_; 28.5 mg FB_1_/kg diet. Table 1 also shows that there were no variations (*p* > 0.05) in the percentage of crude protein, metabolizable energy, digestible energy and the total lipids contents of the formulated feeds.

### 2.1. Effect on Growth Performance

Table 2 shows the results obtained for the zootechnical assessments of the fish fed the various experimental diets. The one way analysis of variance (ANOVA) shows that there were no significant variations (*p* > 0.05) in the body weights of the fish in the treatment groups compared with the body weight of the fish in the control group at the start of the feeding study. However, there were significant variations (*p* < 0.05) in the final weight of the fish in the control group when compared with the final weight of the fish in the treatment groups. The fish fed the control diet (2.0 µg AFB_1_; 3.0 mg FB_1_/kg diet) had the highest final body weight, while the fish fed diet B (19.7 µg AFB_1_; 28.5 mg FB_1_/kg diet) had the lowest weight gain. The Tukey’s post hoc assessment revealed the weight gained by the fish fed diet D (lanthanum chloride 400.0 mg/kg and 19.7 μg AFB_1_/kg + 28.5 mg FB_1_/kg) was significantly (*p* < 0.05) higher than the weight gained by the fish fed diet C (lanthanum chloride 200.0 mg/kg and 19.7 μg AFB_1_/kg + 28.5 mg FB_1_/kg).

The quantity of feed consumed by the experimental fish are shown in Table 2. The one way analysis of variance (ANOVA) shows that the quantity of feed consumed by the fish fed diet A (the control diet) was significantly (*p* < 0.05) more than the quantities of feed consumed by the fish fed the treatment diets (diets B, C and D). The Tukey’s post hoc analysis reveals that the quantities of feed consumed by the fish in the various treatment groups varied significantly (*p* < 0.05) from one another, and that the fish fed diet C (lanthanum chloride 200.0 mg/kg and 19.7 µg AFB_1_ + 28.5 mg FB_1_/kg), consumed the lowest quantity of fed.

In addition, the results obtained for the feed conversion ratio (FCR) are shown in Table 2. The one way analysis of variance (ANOVA) shows that the FCR of the fish fed the control diet (2.0 µg AFB_1_; 3.0 mg FB_1_/kg diet) differed significantly (*p* < 0.05) compared with the FCR of the fish fed the treatment diets (diets B, C and D). The fish fed diet B (19.7 µg AFB_1_ + 28.5 mg FB_1_/kg) had the highest FCR (3.268) while the fish fed diet C had the lowest FCR (1.981). The variations in the FCR of the fish fed diets C (lanthanum chloride 200.0 mg/kg and 19.7 µg AFB_1_ + 28.5 mg FB_1_/kg) and diet D (lanthanum chloride 400.0 mg/kg and 19.7 µg AFB_1_ + 28.5 mg FB_1_/kg) were also not significant (*p* > 0.05).

The results obtained for the feed conversion efficiency of the fish fed the experimental diets are shown in Table 2. The one way analysis of variance (ANOVA) shows the feed conversion efficiency (FCE) of fish fed the control diet differed significantly (*p* < 0.05) compared with the FCE of fish fed the treatment diets (diets B, C and D). Furthermore, Tukey’s post hoc evaluation shows there were no significant variations (*p* > 0.05) in the FCE of the fish fed diet C compared with the FCE of the fish fed diet D. The fish fed the diets containing lanthanum chloride had the highest FCE (50.47), while the fish fed diet B had the lowest FCE (30.60).

The data for the specific growth rate (SGR) shows that the fish fed diet A had the highest SGR (1.195) and the fish fed diet B had the lowest SGR (0.535). The one way analysis of variance (ANOVA) of this data shows that the fish fed diet A (the control diet) had significantly (*p* < 0.05) higher SGR values compared with the fish fed the treatment diets (diets B, C and D). Tukey’s post hoc evaluation reveals there were no significant variation in the SGR of fish fed diet C and diet D.

### 2.2. Effects on Hematology

At 7 days post commencement of the trials (Table 3), the one way analysis of variance (ANOVA) showed that there were significant (*p* < 0.05) decreases in the erythrocytes, leucocytes and the hematocrit values in the fish fed the treatment diets (diets B, C and D) compared with the fish fed the control diet (diet A). Table 3 further shows that the fish fed diet B had the lowest erythrocytes count (1.43 × 10^9^ cells/mm^3^), leucocytes count (1.95 × 10^6^ cells/mm^3^) and hematocrit values (16.24%), while the fish fed the control diet had the highest values for these parameters; there were no clear patterns in these parameters in the fish fed the lanthanum chloride.

In this same period, the one way analysis of variance (ANOVA) also shows that there were significant (*p* < 0.05) variations in the hemoglobin concentration in the fish fed the treatment diets (diets B, C and D) compared with the hemoglobin concentrations of the fish fed the control diet. The Tukey post hoc analysis shows there were no significant (*p* > 0.05) variations in the hemoglobin concentrations of the fish fed diet A (the control diet) and the fish fed diet D.

There was a significant (*p* < 0.05) reduction in the erythrocytes count, the leucocytes count, and the hematocrit volume of the fish fed the treatment diets (diets B, C and D) compared with the corresponding values in fish fed the control diet (diet A) on day 28 (Table 4) and day 56 (Table 5) of the trial. The Tukey’s post assessments reveal there were no significant (*p* > 0.05) variations in the values obtained for fish fed diet C and diet D on day 28 and day 56.

### 2.3. Effects on the Erythrocytic Indices

**Figure 1:**
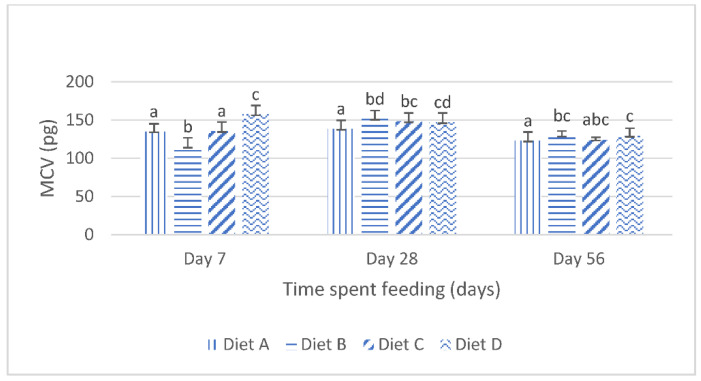
Mean corpuscular volume juvenile *Clarias gariepinus* fed diets amended with lanthanum chloride and mixtures of aflatoxin B1 and fumonisin B1 for 56 days. Bars with different superscripts are significantly different (*p* < 0.05) at specified periods of feeding.

Figure 1 shows the mean corpuscular volume (mcv) of the fish fed the control diet and the contaminated diets amended with lanthanum chloride for 56 days. At day 7 of the feeding study, the mcv of the fish fed the diet contaminated with the mixtures of the AFB_1_ and FB_1_ (diet B) was significantly (*p* < 0.05) reduced compared with the mcv of the fish fed the control diet. The Tukey’s post hoc analysis reveals that, at this same interval, the mcv of the fish fed the mixtures of AFB_1_- and FB_1_-contaminated diets, amended with 400 mg/kg lanthanum chloride, increased significantly (*p* < 0.05) compared with the mcv of the fish fed the control diet (Figure 1). At day 28, the mcv values of the fish fed the treatment diets were significantly higher than those of the fish fed the control diet. The post hoc evaluations showed that there were no significant (*p* > 0.05) variations in the mcv of the fish fed the mycotoxin-contaminated diets (diet B) compared with the mcv of the fish fed the contaminated diet amended with 200 mg/kg lanthanum chloride (diet C). The post hoc assessment further shows that the mcv values obtained for the fish fed diet C were not significantly different (*p* > 0.05) compared with the mcv values of the fish fed diet D. At day 56 of the feeding trial, the one way analysis of variance (ANOVA) showed that the mean mcv of the fish fed the treatment diets were significantly higher (*p* < 0.05) than the mean mcv values of the fish fed the control diet. The post hoc assessment shows that there were no significant variations in the mcv values obtained for the fish fed the treatment diets (*p* > 0.05).

**Figure 2:**
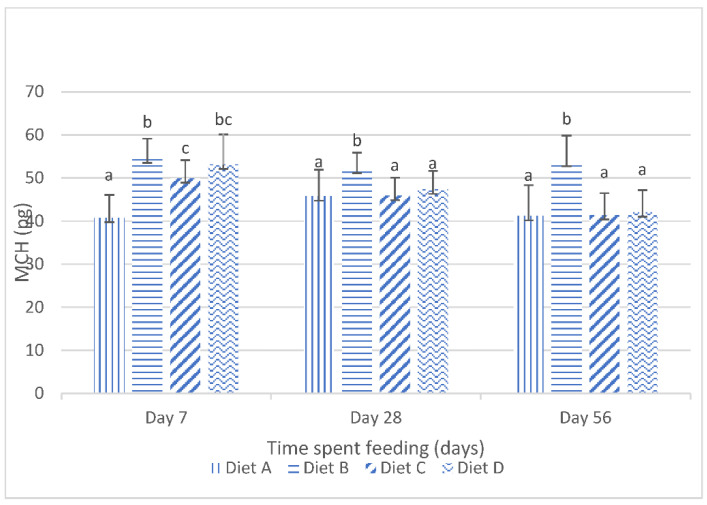
Mean corpuscular hemoglobin of juvenile *Clarias gariepinus* fed diets amended with lanthanum chloride and mixtures of aflatoxin B1 and fumonisin B1 for 56 days. Bars with different superscripts are significantly different (*p* < 0.05) at specified periods of feeding.

The results obtained for the mean corpuscular hemoglobin (mch) of the fish fed the experimental diets are shown in Figure 2. They show that, at day 7 of the feeding study, there were significant (*p* < 0.05) increases in the mch values of the fish fed the treatment diets compared to the mch values of the fish fed the control diet. Figure 2 also shows the significant (*p* < 0.05) reduction in the mch values in the fish fed the contaminated diets, amended with lanthanum chloride, at day 28 and day 56 of the feeding trial. The post hoc assessment shows that there were no significant (*p* > 0.05) variations in the mch values of the fish fed the contaminated diets, amended with 200 mg/kg lanthanum chloride and 400 mg/kg lanthanum chloride, and the control diet at these sampling intervals.

**Figure 3:**
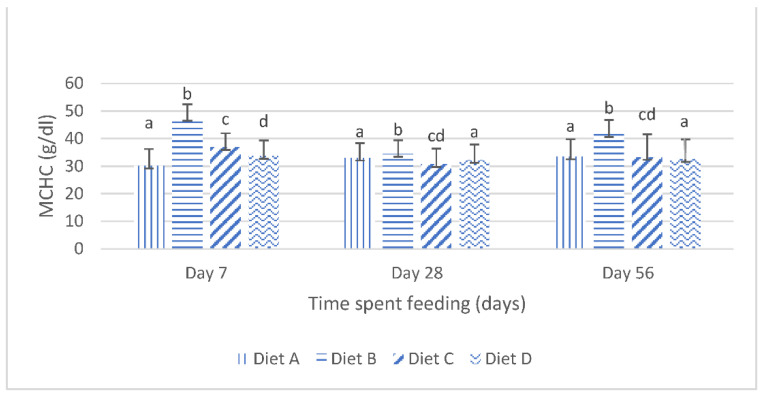
Mean corpuscular hemoglobin concentration of juvenile *Clarias gariepinus* fed diets amended with lanthanum chloride and mixtures of aflatoxin B1 and fumonisin B1 for 56 days. Bars with different superscripts are significantly different (*p* < 0.05) at specified periods of feeding.

The mean corpuscular hemoglobin concentration (mchc) of the fish fed the experimental diets are shown in Figure 3. It shows that, at day 7 of the feeding, the mchc values of the fish fed the diets contaminated with mixtures of AFB_1_ and FB_1_ were significantly (*p* < 0.05) increased compared with the mchc values of the fish fed the contaminated diets amended with lanthanum chloride. There were no significant variations in the mchc values of the fish fed the contaminated diet B and the fish fed the contaminated diet C, amended with 200 mg/kg lanthanum chloride, at days 28 and day 56; furthermore, the mchc values of the fish fed the contaminated diet D, amended with 400 mg/kg lanthanum chloride, was not significantly (*p* > 0.05) different from the mchc values of the fish fed the control diet, at days 28 and 56 of the study (Figure 3).

The results obtained for the serum biochemistry evaluations are shown in Table 6 and Table 7. At 7 days post commencement of the trial, there were no significant variations (*p* > 0.05) in the serum total protein concentrations of the fish fed diet A (the control diet) compared with those of the fish fed the treatment diets (diets B, C and D). The serum albumin concentration of the fish fed diet D (the contaminated diet amended with 400 mg/kg lanthanum chloride) was significantly (*p* < 0.05) higher compared with those of the fish fed diet A (the control diet). The Tukey’s post hoc assessment shows the variations in the serum albumin concentrations of fish fed diet C and diet D were not significant (*p* > 0.05). Table 6 also shows there were significant increases (*p* < 0.05) in the serum transaminase activities of fish fed the treatment diets compared with those of fish fed the control diets. The Tukey’s post hoc assessment shows that there were no significant variations (*p* > 0.05) in the alanine amino transferase and aspartate amino transferase values of the fish fed 200 mg/kg and 400 mg/kg lanthanum chloride.

At this same time interval, the serum creatinine, urea and uric acid concentrations of the fish fed the experimental diets (diets B, C and D) were significantly (*p* < 0.05) higher compared with those of the fish fed the control diet; Tukey’s post hoc evaluations at this interval also show the serum concentrations of the creatine, urea and uric acid obtained for the fish fed the diets amended with 200 mg/kg (diet C) and 400 mg/kg lanthanum chloride (diet D) were significantly (*p* < 0.05) lower than those obtained for the fish fed only the contaminated diet (diet B); furthermore, the differences obtained in these values for the fish fed 200 mg/kg lanthanum chloride and 400 mg/kg lanthanum chloride were not significant (*p* > 0.05).

The results obtained for the serum biochemical evaluations at day 56 of the feeding trial are shown in Table 7. It shows that the serum total protein concentrations of the fish fed the treatment diets were significantly (*p* < 0.05) higher compared with those of the fish fed the control diet. The Tukey’s post hoc assessment reveals that there were no significant (*p* > 0.05) variations in the serum total protein concentration of the fish fed diet C and diet D. Table 7 also shows that the serum albumin concentration of the fish fed the control diet differed significantly (*p* < 0.05) compared with those of the fish fed diets B, C and D.

The serum globulin concentrations of the fish fed diets B, C and D were significantly (*p* < 0.05) higher compared with those of the fish fed diet A (the control diet) at 56 days post commencement of the trial (Table 7). The Tukey’s post hoc evaluation reveals the difference in the serum globulin concentrations of the fish fed diet B and diet D, at 56 days of the feeding trial, were not significant (*p* > 0.05).

The serum transaminases (alanine aminotransferase and aspartate aminotransferase activities), and the alkaline phosphatase activities increased significantly (*p* < 0.05) in the fish fed diets B, C and D compared with their corresponding values in the fish fed the control diet (Table 7). The Tukey’s post hoc evaluation shows the serum alanine aminotransferase (ALT) activities of the fish fed diet B, and diet D differed significantly (*p* < 0.05) at 56 days of feeding, with the fish fed diet B having the lowest ALT activity (25.00 u/L) and the fish fed diet D having the highest ALT activity (33.51 u/L).

The serum concentrations of creatinine, urea and uric acid and the serum activity of lactate dehydrogenase (LDH) increased significantly (*p* < 0.05) in the fish fed the treatment diets (diets B, C and D) compared to their corresponding values in the fish fed the control diet (diet A) at 7 days of feeding (Table 6) and at 56 days of feeding (Table 7).

## 3. Discussion

The water quality parameters of the culture tanks in the present study were determined to be within the range recommended for the culture of clariid catfishes [39]; thus, may not have contributed to the pathophysiological observations recorded in this study. The proximate and mycotoxin (AFB_1_ + FB_1_) content of the feed was not altered by the addition of the lanthanum chloride; furthermore, the AFB_1_ and FB_1_ content of the final feed was higher than the respective concentrations of the purified mycotoxins introduced into the diets at formulation. This is an expected result, as it has been previously noted that agricultural products are often contaminated with various mycotoxins, and that these mycotoxins occur at varying concentrations; hence, the difference in the AFB_1_ and FB_1_ contents of the produced diets reflects the concentrations of these mycotoxins in the agricultural materials used in the production of the feed [9,43].

The fish fed diet A (the control diet) consumed the most quantity of feed, while the fish fed diet B (19.7 µg AFB_1_ + 28.5 mg FB_1_/kg) consumed the lowest quantities of fed. This is an expected result, as mycotoxins, especially aflatoxins, are reported to cause a reduction in feeding, or an outright feed refusal, with a consequent decrease in the performance in the animals [41,44]. This agrees with the findings of [32], who observed that dietary rare earth elements improve the body weight gain and feed conversion ratio, without increasing feed intake. The results of the present study also show that the fish fed diets containing 400 mg/kg lanthanum chloride consumed more feed compared with the fish fed 200 mg/kg lanthanum chloride; thus, indicating that the feed consumption increased with the dietary concentration of lanthanum chloride.

The fish fed the diets containing AFB_1_ and FB_1_ exhibited the lowest weight gain. This is similar to the findings of our earlier study [39], where poor growth performance was recorded in the juvenile *Clarias gariepinus* catfish when fed diets amended with doses of mixtures AFB_1_ and FB_1._ The aflatoxins are reported to cause gastrointestinal dysfunctions marked by significant changes in the gut morphology, reduced digestive ability and a disruption of the digestive enzymes and intestinal innate immunity [23]. The fumonisins are reported to negatively influence growth performance by their abilities to interfere with cellular growth and cell–cell interactions [20,45]. The poor growth recorded for fish when fed diets containing mixtures of AFB_1_ and FB_1_ may be as a consequence of the combined activities of the two mycotoxins.

The fish fed diet D (400 mg/kg lanthanum chloride) exhibited a superior weight gain compared with the fish fed diet C (200 mg/kg lanthanum chloride), suggesting that the weight gain in the juvenile *Clarias gariepinus,* fed diets contaminated with mixtures of aflatoxin B1 and fumonisin B1, was influenced by the concentration of the dietary inclusion of lanthanum chloride. The exact mechanisms of the growth promotion by lanthanum chloride are yet to be described. It is, however, reported that lanthanum chloride may promote weight gain in animals by improving the utilization of dietary nutrients, such as total energy, crude protein and fat [46]. It is also reported that dietary lanthanum chloride increases the secretion of gastric juices in the exposed animals [47]; thus, the increased weight gain observed in the present study may be a function of the increased activities of gastric enzymes in the exposed fish [47] and, since the fish fed the diets containing lanthanum chloride at 400 mg/kg diet consumed more feed compared with those fed the diets containing lanthanum chloride at 200 mg/kg diet, it is therefore reasonable for them to gain better weight.

The fish fed diet B (19.7 µg AFB_1_ + 28.5 mg FB_1_/kg diet) exhibited the highest (3.268), and the lowest feed conversion ratio (30.60 ± 1.60) compared with the fish fed the other diets. This is a result of the deleterious effects of the mixed mycotoxins in the diets [13,40,48,49]. The feed conversion ratio and the feed conversion efficiency were significantly improved by the addition of the lanthanum chloride into the diets. The results of the present study further show that fish fed diet C (lanthanum chloride 200 mg/kg diet), exhibited the lowest (1.981 ± 0.07) feed conversion ratio. Hence, the lanthanum chloride at 200 mg/kg inclusion rates produced the best nutrient utilization in the juvenile *Clarias gariepinus* fed diets contaminated with mixtures of aflatoxin B1 and fumonisin B1.

The evaluations of the hematological parameters of the fish are required in the physiological assessment of the effects of exposure to sub-chronic concentrations of contaminants [50] and/or the physiological response to the dietary intake of essential nutrients [51]. This is because the determination of the erythrocytes count, the hematocrit values, and the hemoglobin concentration in the fish aids in the assessment and prognostication of anemias [52].

The results of the present study show significant decreases in the erythrocytes counts, the hematocrit values and the hemoglobin concentrations of the fish fed the diets contaminated with mixtures of AFB_1_ and FB_1_ compared with the corresponding values for the fish fed the control diets at days 7, 28 and 56 of the trial. This is similar to the findings of [8], who reported a disruption in the protein digestion and absorption in Nile tilapia following dietary exposures to AFB_1_.

There were no significant variations in the erythrocytes counts, the hematocrit values and the hemoglobin concentrations of the fish fed the low (200 mg/kg) or high (400 mg/kg) concentrations of lanthanum chloride; however, the fish fed the mycotoxin-contaminated diets amended with lanthanum chloride exhibited significantly higher erythrocytes counts, hematocrit values and hemoglobin concentrations compared with their corresponding values in the fish fed diets contaminated with the mixtures of AFB_1_ + FB_1_ only. This indicated that dietary lanthanum chloride may ameliorate the depression of erythropoiesis induced by dietary exposures to mixtures of AFB_1_ and FB_1_. This finding may be a result of the increases in feed consumption and improved utilization of dietary nutrients [53,54], or due to the anti-oxidative effects of lanthanum chloride, wherein lanthanum chloride is able to protect the oxidation of dietary fatty acids, such as omega-3 fatty acids, thereby making it more available and/or enhancing their absorption [55].

There were significant and sustained leukocytopenia in the fish fed the diets contaminated with AFB_1_ and FB_1_ throughout the duration of the study. This agrees with the reports [21,45,48,49], where it was reported that the dietary mycotoxins elicit a suppression of the immune response of exposed animals. Furthermore, the fish fed the diets contaminated with AFB_1_ and FB_1_ and containing lanthanum chloride exhibited significantly higher leucocytes counts compared with the fish fed diets contaminated AFB_1_ and FB_1_ alone, indicating lanthanum chloride may have some ameliorative effect on the leucocytes counts of juvenile *Clarias gariepinus* fed diets contaminated with mixtures of AFB_1_ and FB_1._ The mechanism for these immunoprotective effects may not be unconnected with the antioxidant activities of lanthanum chloride [53,55] and/or the increased nutrient absorption and utilization effects of lanthanum chloride [56,57].

Although the results of the present study show that the dietary lanthanum chloride elicited significant changes in the erythrocytic indices (the mean corpuscular volume, the mean corpuscular hemoglobin values and the mean corpuscular hemoglobin concentration) of juvenile *Clarias gariepinus* fed with the contaminated diets, the observed changes were well within the scope of the hematological reference intervals for juvenile *Clarias gariepinus* [39]. It is probable that these changes may have been more pronounced if the duration of the study had extended beyond the 56 days duration, as it is generally reported that the effects of dietary exposures to mycotoxins are dependent on the duration of the exposure and the concentration of the mycotoxins [5,13,20].

The serum total proteins, consisting of the albumin and globulin concentrations, provide critical information reflecting the functional statuses of various organs and/or systems; since they are involved in the specific immune responses of the fish and participate in the maintenance of the acid-base balance [58,59], the serum proteins are also involved in the protection of the cellular integrity of cells, such as the erythrocytes, hepatocytes and the nephrocytes [50]. The serum total proteins also provide an easy and readily available source of energy in emergencies, such as that obtained in situations of feed deprivation [60,61,62].

The serum total proteins increase in cases of generalized chronic inflammation and in inflammatory disorders affecting the liver and the kidneys [60]. The present study was marked by hyperproteinemia (observed 56 days post dietary exposure) in the fish fed the diets contaminated with mixtures of AFB_1_ and FB_1_; this is an expected result as both of the mycotoxins have been reported to elicit hepatic and nephrotic syndromes in exposed fish [45,63]. There were no significant differences in the serum total protein of the fish fed the diets amended with lanthanum chloride or bentonite clay. This may be the consequence of ingested free mycotoxins, especially of the fumonisins [64].

The serum albumins are produced in the liver. Therefore, the synthetic capacity of the liver (which is an estimate of the protein losing nephropathy) may be estimated by the determination of the serum albumin concentration [65]. According to [66], malnutrition, increased protein catabolism, enteropathy and/or chronic nephropathy are marked by a reduced serum albumin concentration (termed hypoalbuminemia). The fish fed the diets contaminated with the mixtures of AFB_1_ and FB_1_ exhibited significantly elevated serum albumin concentrations. These may be a result of the combined effects of AFB_1_ and FB_1_ [63]. As observed for the serum total proteins, the inclusion of the lanthanum chloride in the diets elicited a marginal but significant reduction in the serum albumin concentration compared with those of the fish fed the diets contaminated with only the mixtures of AFB_1_ and FB_1_. This may indicate that lanthanum chloride may have some hepatoprotective and nephroprotective properties in juvenile *Clarias gariepinus* fed diets contaminated with mixtures of AFB_1_ and FB_1._

The hepatic enzymes (transaminase and alkaline phosphatase) are liberated into the serum in situations of hepatocellular or cholestatic liver injuries [67]. In hepatopathies, such as those seen in hepatocellular degenerations, aspartate aminotransferase (AST) and alanine aminotransferase (ALT) are liberated into the serum, while alkaline phosphatase (ALP) is liberated into the serum in hepatic cholestasis [66]. The results of the present study shows significant elevations in the serum activities of these enzymes in the fish fed the diets containing the mixtures of AFB_1_ and FB_1_. The highest values for the AST, ALT and ALP were obtained in the fish fed diet B (contaminated with AFB_1_ and FB_1_). The AST, ALT and the ALP activities were significantly lower in the fish fed the diets containing lanthanum chloride and bentonite clay, compared with the corresponding values in the fish fed AFB_1_- and FB_1_-contaminated diets only. The fumonisins are reported to cause cellular rupture and necrosis by the inhibition of mitochondrial respiration and the complete deregulation of calcium homeostasis [68]; meanwhile, by its ability to preferentially bind to calcium, the lanthanum chloride may prevent tissue necrosis via this mechanism and, hence, reduce the elaboration of these enzymes into the serum, indicating some erythrocyte, hepatic and kidney protective effects of the dietary lanthanum chloride [24,69].

The serum creatinine concentration is increased significantly in the skeletal muscle necrosis and/or atrophy, as well as in chronic nephropathies [58,70]. In the present study, there were significant elevations of the serum creatinine concentration in the fish fed the diets containing the mixtures of AFB_1_ and FB_1_. The highest values for the serum creatinine concentration were obtained in the fish fed diet B (contaminated with AFB_1_ and FB_1_), indicating significant skeletal muscle necrosis and/or atrophy, as well as probable chronic nephropathy [66]. The serum creatinine concentrations of the fish fed AFB_1_ and FB_1_-contaminated diets, containing lanthanum chloride or bentonite clay, were significantly lower compared with those of the fish only fed the AFB_1_- and FB_1_-contaminated diets. This may be indicative of some liver and kidney protective effects of the lanthanum chloride in the *Clarias gariepinus* fed the diets contaminated with the mixtures of AFB_1_ and FB_1_ [24,71].

The serum urea nitrogen and the uric acid concentrations are critical analytes required in the assessments of the functional status of the kidney [67]. It is reported that decreases in the blood urea nitrogen concentrations are usually observed in hepatic insufficiencies and in cases of malnutrition, while an increased blood urea nitrogen concentration is commonly reported in renal disease, shock and in cardiac insufficiencies [71,72]. The results obtained from the present study show the serum urea and uric acid concentrations of the fish fed the diets contaminated with the mixtures of AFB_1_ and FB_1_ were significantly higher than those of the fish fed the control diet, indicating a significant impact on the kidneys [8,41,69]. The fish fed the diets contaminated with the mixtures of AFB_1_ and FB_1_ containing lanthanum chloride exhibited significantly lowered serum urea and uric acid concentrations compared to the fish fed the diets contaminated with only mixtures of AFB_1_ and FB_1_, indicating that the dietary lanthanum chloride in juvenile *Clarias gariepinus* may have some ameliorating effects on the AFB_1_- and FB_1_-induced kidney toxicities [70,73].

Lactate dehydrogenase (LDH) is an enzyme found in several tissues/organs (such as the muscles, liver, heart, kidneys and the blood vessels. It catalyzes the reversible transformation of pyruvate into lactate [74]. The increased serum activity of LDH is indicative of degenerative changes in any of the aforementioned tissues/organs [66,67]. The results of our study show that the serum LDH activity of the fish fed the diets contaminated with only mixtures of AFB_1_ and FB_1_ were significantly higher compared with those of the fish fed the control diet, indicating a significant impact on the kidneys and/or the other aforementioned organs [67,70]. The fish fed the diets contaminated with the mixtures of AFB_1_ and FB_1_ containing lanthanum chloride exhibited significantly lowered serum urea and uric acid concentrations compared to the fish fed the diets contaminated with only mixtures of AFB_1_ and FB_1_, indicating that the dietary lanthanum chloride in juvenile *Clarias gariepinus* may have some ameliorating effects on the toxicities of the mixtures of AFB_1_ and FB_1_ [40,75].

## 4. Conclusions

Under the present culture conditions, the dietary lanthanum chloride at 200 or 400 mg/kg feed could promote the growth performance, nutrient utilization and ameliorate the hematological and serum biochemical derangements produced by the dietary exposures to the mixtures of AFB_1_ and FB_1_ in the juvenile *Clarias gariepinus* catfish. A further study is needed to determine and confirm the exact dietary concentration of lanthanum chloride needed for the optimization of the growth and health performance of this fish species, under the challenge of inadvertent dietary exposures to mixtures of aflatoxin B1 and fumonisin B1.

## 5. Materials and Methods

### 5.1. Experimental Fish and Experimental Design

Three hundred and sixty (360) juvenile *C. gariepinus* catfish were acquired from a commercial catfish hatchery. The fish were allowed to acclimatize to laboratory conditions for 21 days before the commencement of the experiment.

Fifteen (12) 1000 L capacity tanks retrofitted with water inflow and outflow devices were divided into five groups (each consisting of a triplicate set of tanks, with each tank containing 30 juvenile *C. gariepinus catfish*), as described in [75].

The experiment adopted a complete randomized design with a triplicate of each treatment. Four groups, consisting of one control and three treatment groups, were used for this study. Group A (control group, were fed the control diet, i.e., no lanthanum chloride, no mycotoxin); Group B (treatment 1, were fed the basal diet amended with mixtures of AFB_1_ and FB_1_ at an inclusion rate of 19.7 μg AFB_1_/kg diet and 28.5 mg FB_1_/kg diet); Group C (treatment 2, were fed the basal diet amended with lanthanum chloride 200.0 mg/kg diet and mixtures of AFB_1_ and FB_1_ at an inclusion rate of 19.7 μg AFB_1_/kg diet and 28.5 mg FB_1_/kg diet); Group D (treatment 3, were fed the basal diet amended with lanthanum chloride 400.0 mg/kg diet and mixtures of AFB_1_ and FB_1_ at an inclusion rate of 19.7 μg AFB_1_/kg diet and 28.5 mg FB_1_/kg diet). The groups of fish were fed any of the diets A, B, C or D in triplicates for 56 days.

### 5.2. Experimental Feeds

The experimental feeds were produced at the University of Abuja feed mill, following the procedures reported by [39], with slight adjustments. The basal diet was formulated using the following ingredients (fish meal 19%, soybean cake 37%, maize 32.25%, palm oil 1.0%, fish oil 6.0%, starch binder 2.0%, vitamin/mineral premix 0.5%, bone meal 1.0%, salt 0.25%), to meet the nutritional requirements of the juvenile clariid fish [37].

The mixtures of the AFB_1_ and FB_1_ diets were produced by adding 1 mg crystalline AFB_1_ (Sigma Chemicals, St Louis, MO, USA) to 1 mL chloroform (to produce 1 mg: 1000 μL aliquot of AFB_1_). The quantity of the solution required to produce the chosen concentration of the AFB_1_ in the mixed mycotoxin diets was then pipetted using an automated adjustable pipette into 100 mL volumetric flasks. This volume was then made up to the 100 mL mark with methanol. The FB_1_ contents of the respective diets were added by careful measurements of the desired quantities of FB_1_ from a pre-produced aliquot of 1 g FB_1_ dissolved in 1000 μL of acetonitrile-water (*v*/*v*) (resulting in 1 μL:1 mg solution of fumonisin B1).

The ingredients for the basal diets were weighed, completely mixed, and added to the liquid mixture of AFB_1_ and FB_1_ (i.e., 17.0 µg AFB_1_; 23.0 FB_1_/kg diet)_,_ 200.0 mg lanthanum chloride, 400.0 mg lanthanum chloride or 400 mg/kg. Diet A (control 0.0 μg AFB_1_/kg + 0.0 mg FB_1_/kg diet); diet B (17.0 µg AFB_1_; 23.0 FB_1_/kg diet); diet C (lanthanum chloride 200.0 mg/kg and 17.0 µg AFB_1_; 23.0 FB_1_/kg diet) and diet D (lanthanum chloride 400.0 mg/kg and 17.0 µg AFB_1_; 23.0 FB_1_/kg diet).

The mixtures were subsequently blended and placed in a hot air oven for the methanol to evaporate. These were then pelletized with an extruder pelletizer, after the addition of weighted portions of the starch binders. The mycotoxins content of the compounded diets were then analyzed using the multi-mycotoxin LC-MS/MS method [76,77] and, thereafter, individually packed in airtight polyethylene bags and stored in a deep freezer (at 2–4 °C) until use. According to the results obtained following the mycotoxin assessments of the produced diets, the AFB_1_ and FB_1_ concentrations of the produced experimental diets were consequently adjusted, as shown in Table 1.

At time points day 7, 28 and day 56, five (5) fish were randomly selected (by the aid of a handheld sampling net) from each tank for hematological determinations. The sampled fish were weighed, length measured and then bled via caudal veni-puncture, using a 23 G needle fitted on a 5 mL syringe pre-rinsed with ethylene diamine tetra acetic (EDTA) solution (for hematological assessments), and without EDTA (for serum biochemical determinations).

### 5.3. Hematological Analysis

The hematological parameters, such as the hemoglobin concentration (Hb), total erythrocytes count (RBC) and total leucocytes count (WBC), packed cell volume (PCV), mean corpuscular hemoglobin (MCH), mean corpuscular hemoglobin concentration (MCHC) and mean corpuscular volume (MCV), were analyzed.

The Hb concentration was determined by the cyanmethemoglobin method [50]. A Neubauer hemocytometer was used for the determination of the total red and white blood corpuscle count, by using Natt and Herricks dilution fluid. The cell count was estimated as per [78], using the following formulae:

RBC count = number of cells counted × dilution factor × depth of chamber/area counted

where the dilution factor is one in 200; the depth is 1/10 mm; and the area counted is 80/400 = 1/5 sq.

WBC count = number of cells counted × blood dilution× chamber depth/number of chambers counted:

WBC/mm^3^ = total white blood cells × 200 × 10/4

The hematocrit (PCV) was determined by the following methods of [50] briefly, where micro-hematocrit capillaries were centrifuged at 12,000 *g* for 5 min in a micro-hematocrit centrifuge (Ocean Med, London, UK). The erythrocytic indices were determined mathematically, following the methods of [58].

### 5.4. Serum Biochemical Parameters

The serum was obtained from the blood (collected without anticoagulant), and kept in the narrow-bored glass test tubes after centrifugation at 5000× *g* for 5 min in a laboratory centrifuge (Uniscope model SM 112, Surgifield Medicals, Devon, UK. After centrifugation, the liquid fraction (the serum) was eluted from the top of the tube, using a clean and sterile 1 mL syringe. The collected serum was then transferred into a fresh Eppendorf tube and stored at −20 °C until used for biochemical analyses.

From the serum, the following biochemical parameters were determined: total protein and albumin (in g dL^−1^) using the methods of [17]; alanine amino transaminase and aspartate aminotransferase (UL^−1^), alkaline phosphatase (UL^−1^), lactase dehydrogenase (UL^−1^), creatinine kinase (µmol/L) following the methods of [67]. The serum urea (mg dL^−1^) and uric acid concentrations (µmol/L)) were determined, using the methods of [79]. The serum biochemistry evaluations were carried out, using a laboratory spectrophotometer (SpectrumLab 750, Huddinge, Sweden) and Dialab serum diagnostic kits (Dialab, Neudorf, Austria). The globulin values were calculated mathematically by subtracting the albumin values from the values obtained for serum total protein. All of the serum biochemical parameters were obtained by following strictly the guidelines of the manufacturers of the diagnostic kits.

### 5.5. Zootechnical Evaluations

The fish in each group were fed the respective feed at 5% of the biomass (the feed was divided into two portions, given at 0800 h and at 1800 h) daily. All of the unconsumed feed was siphoned, dried and weighed in order to determine the exact amount of feed consumed per group. At the set time points, the fish were individually weighed (to the nearest 0.00, using a sensitive laboratory scale) and their length measured (to the nearest 0.00 cm using a ruler). At the close of the feeding trial, the following growth indices were determined mathematically according to [80]:

(a) Growth rate (g/d) = W_f_ − W_i_/T_f_ − T_i_
  Where W_f_ = final weight
  W_i_ = initial weight
  T_f_ − T_i_ = Time (in days) spent feeding between W_f_ and W_i_;
(b) Specific growth rate (%/day) = 100 × [(ln W_f_ − ln W_i_)/(T_f_ − T_i_)];
(c) Feed conversion ratio (FCR) = weight of feed consumed (g)/W_f_ − W_i_ (W_f_ = final weight; W_i_ = initial weight);
(d) Feed conversion efficiency (%) = [W_f_ − W_i_ weight of feed consumed (g)] × 100

### 5.6. Statistical Evaluation

Data were expressed as the mean ± Standard Deviation of the mean of each group (*n* = 30) and analyzed by a one way analysis of variance (ANOVA). The variant means were then separated by the Duncan’s multiple range post hoc test using the Statistical Package of Social Sciences (SPSS) for Windows version 20.0 (IBM Corporation, Armonk, USA). Differences in the means were considered significant at *p* < 0.05.

## Figures and Tables

**Table 1:** Ingredients and Proximate Composition of Diets Amended with Lanthanum chloride, Bentonite clay and Mixtures of Aflatoxin B_1_ and Fumonisin B_1_.

*Parameter*	*Diet A*	*Diet B*	*Diet C*	*Diet D*
*^¥^* *Fish meal (%)*	19.0	19.0	19.0	19.0
*Soybean cake (%)*	38.00	38.00	38.00	38.00
*Maize (%)*	32.22	32.24	32.23	32.23
*Palm oil (%)*	1.00	1.00	1.00	1.00
*Fish oil (%)*	6.00	6.00	6.00	6.00
*Vit/min premix (%)*	0.50	0.50	0.50	0.50
*Bone meal (%)*	1.00	1.00	1.00	1.00
*NaCl (%)*	0.23	0.23	0.23	0.23
*LC (mg/kg)*	0.0	0.0	200.00	400.00
*AFB_1_ (μg/kg): FB_1_ (mg/kg)*	2.0; 3.0	19.7; 28.5	19.7; 28.5	19.7; 28.5
*^Ʊ^ Starch binder (%)*	2.00	2.00	2.00	2.00
*Crude protein (%)*	40.04	40.00	40.02	40.01
*Gross energy (kj/g)*	20.00	20.01	19.97	20.02
*Digestible energy (kj/g)*	12.00	12.04	12.01	12.07
*Total lipids (%)*	9.0	9.0	9.0	9.0
*Moisture (%)*	2.27	2.33	2.33	2.33
*Ash (%)*	9.65	9.70	9.71	9.65

^¥^ Fish meal of 72% crude protein; ^Ʊ^ Cassava starch binder; LC = Lanthanum chloride; BC = Bentonite clay. Diet A (control 2.0 μg AFB_1_/kg + 3.0 mg FB_1_/kg); Diet B (19.7 μg AFB_1_/kg + 28.5 mg FB_1_/kg); Diet C (lanthanum chloride 200.0 mg/kg and 19.7 μg AFB_1_/kg + 28.5 mg FB_1_/kg); Diet D (lanthanum chloride 400.0 mg/kg and (19.7 μg AFB_1_/kg + 28.5 mg FB_1_/kg).

**Table 2:** Growth response of juvenile *Clarias gariepinus* catfish fed diets amended with lanthanum chloride and mixtures of aflatoxin B1 and fumonisin B1 for 56 days.

	*Diet A (Control)*	*Diet B*	*Diet C*	*Diet D*
*Mean initial body weight (g)*	85.04 ± 1.17 ^a^	85.00 ± 0.21 ^a^	85.02 ± 1.21 ^a^	85.09 ± 1.01 ^a^
*Mean final body weight (g)*	168.0 ± 4.99 ^a^	116.2 ± 2.07 ^b^	131.4 ± 6.35 ^c^	138.5 ± 3.19 ^d^
*Mean weight gained (g)*	81.00 ± 1.11 ^a^	31.5 ± 1.12 ^b^	47.90 ± 1.09 ^c^	51.2 ± 1.25 ^d^
*Feed fed (g)*	197.38 ± 1.42 ^a^	102.94 ± 1.11 ^b^	94.91 ± 1.13 ^c^	105.04 ± 1.08 ^d^
*FCR*	2.437 ± 0.11 ^a^	3.268 ± 0.05 ^b^	1.981 ± 0.07 ^c^	2.052 ± 0.07 ^c^
*FCE*	41.04 ± 2.97 ^a^	30.60 ± 1.66 ^b^	50.47 ± 1.14 ^c^	49.21 ± 1.12 ^d^
*SGR (%/day)*	1.19 ± 0.03 ^a^	0.54 ± 0.01 ^b^	0.77 ± 0.01 ^cd^	0.83 ± 0.66 ^cd^
*Survival (%)*	100.00	100.00	100.00	100.00

Diet A (control 2.0 μg AFB_1_/kg + 3.0 mg FB_1_/kg); Diet B (19.7 μg AFB_1_/kg + 28.5 mg FB_1_/kg); Diet C (lanthanum chloride 200.0 mg/kg and 19.7 μg AFB_1_/kg + 28.5 mg FB_1_/kg); Diet D (lanthanum chloride 400.0 mg/kg and 19.7 μg AFB_1_/kg + 28.5 mg FB_1_/kg). Rows with different superscripts are significantly different (*p* < 0.05).

**Table 3:** Hematological profile of juvenile *Clarias gariepinus* catfish fed diets amended with lanthanum chloride and mixtures of aflatoxin B1 and fumonisin B1 for 7 days.

*Parameter*	*Diet A (Control)*	*Diet B*	*Diet C*	*Diet D*
*Erythrocytes (10^9^ cells/mm^3^)*	2.21 ± 0.01 ^a^	1.43 ± 0.04 ^b^	1.68 ± 0.01 ^c^	1.60 ± 0.02 ^d^
*Leucocytes (10^6^ cells/mm^3^)*	2.30 ± 01.01 ^a^	1.95 ± 0.01 ^b^	2.04 ± 0.03 ^c^	2.10 ± 0.01 ^d^
*Hematocrit (%)*	29.85 ± 0.12 ^a^	16.42 ± 0.36 ^b^	22.76 ± 1.88 ^c^	25.19 ± 1.17 ^d^
*Hemoglobin concentration (g/dl)*	9.00 ± 0.59 ^a^	7.79 ± 0.64 ^b^	8.39 ± 0.51 ^c^	8.50 ± 0.39 ^a^

Diet A (control 2.0 μg AFB_1_/kg + 3.0 mg FB_1_/kg); Diet B (19.7 μg AFB1/kg + 28.5 mg FB1/kg); Diet C (lanthanum chloride 200.0 mg/kg and 19.7 μg AFB_1_/kg + 28.5 mg FB_1_/kg); Diet D (lanthanum chloride 400.0 mg/kg and 19.7 μg AFB1/kg + 28.5 mg FB_1_/kg). Rows with different superscripts are significantly different (*p* < 0.05).

**Table 4:** Hematological profile of juvenile *Clarias gariepinus* catfish fed diets amended with lanthanum chloride and mixtures of Aflatoxin B1 and fumonisin B1 for 28 days.

*Parameter*	*Diet A(Control)*	*Diet B*	*Diet C*	*Diet D*
*Erythrocytes (10^9^ cells/mm^3^)*	2.18 ± 0.07 ^a^	1.21 ± 0.06 ^b^	1.72 ± 0.01 ^c^	1.69 ± 0.01 ^c^
*Leucocytes (10^6^ cells/mm^3^)*	2.26 ± 0.01 ^a^	1.14 ± 0.01 ^b^	2.11 ± 0.02 ^c^	2.13 ± 0.01 ^d^
*Hematocrit (%)*	30.17 ± 1.39 ^a^	18.31 ± 4.12 ^b^	25.49 ± 1.09 ^c^	24.83 ± 1.00 ^d^
*Hemoglobin concentration (g/dl)*	9.97 ± 0.59 ^a^	6.31 ± 1.47 ^b^	7.83 ± 0.98 ^cd^	8.00 ± 0.81 ^cd^

Diet A (control 2.0 μg AFB_1_/kg + 3.0 mg FB_1_/kg); Diet B (19.7 μg AFB1/kg + 28.5 mg FB1/kg); Diet C (lanthanum chloride 200.0 mg/kg and 19.7 μg AFB_1_/kg + 28.5 mg FB_1_/kg); Diet D (lanthanum chloride 400.0 mg/kg and 19.7 μg AFB1/kg + 28.5 mg FB_1_/kg). Rows with different superscripts are significantly different (*p* < 0.05).

**Table 5:** Hematological profile of juvenile *Clarias gariepinus* catfish fed diets amended with lanthanum chloride and mixtures of Aflatoxin B1 and fumonisin B1 for 56 days.

*Parameter*	*Diet A (Control)*	*Diet B*	*Diet C*	*Diet D*
*Erythrocytes (10^9^ cells/mm^3^)*	2.44 ± 0.01 ^a^	1.25 ± 0.06 ^b^	2.06 ± 0.01 ^c^	1.99 ± 0.01 ^d^
*Leucocytes (10^6^ cells/mm^3^)*	2.26 ± 0.01 ^a^	1.09 ± 0.01 ^b^	2.03 ± 0.02 ^c^	2.01 ± 0.01 ^d^
*Hematocrit (%)*	30.00 ± 2.03 ^a^	16.18 ± 6.04 ^b^	25.61 ± 2.39 ^c^	25.70 ± 4.05 ^c^
*Hemoglobin concentration (g/dl)*	10.05 ± 0.16 ^a^	6.72 ± 1.39 ^b^	8.53 ± 0.74 ^c^	8.37 ± 0.55 ^c^

Diet A (control 2.0 μg AFB_1_/kg + 3.0 mg FB_1_/kg); Diet B (19.7 μg AFB_1_/kg + 28.5 mg FB_1_/kg); Diet C (lanthanum chloride 200.0 mg/kg and 19.7 μg AFB_1_/kg + 28.5 mg FB_1_/kg); Diet D (lanthanum chloride 400.0 mg/kg and 19.7 μg AFB_1_/kg + 28.5 mg FB_1_/kg). Rows with different superscripts are significantly different (*p* < 0.05).

**Table 6:** Serum biochemical profile of juvenile *Clarias gariepinus* catfish fed diets amended with lanthanum chloride and mixtures of aflatoxin B1 and fumonisin B1 for 7 days.

*Parameter*	*Diet A (Control)*	*Diet B*	*Diet C*	*Diet D*
*Total Protein (g/dL)*	6.01 ± 1.08 ^a^	6.00 ± 1.27 ^a^	5.94 ± 1.12 ^a^	6.01 ± 1.13 ^a^
*Albumin (g/dL)*	3.81 ± 0.23 ^a^	3.93 ± 0.17 ^a^	4.10 ± 0.11 ^ab^	4.17 ± 0.88 ^b^
*Globulin (g/dL)*	2.20 ± 0.01 ^a^	2.07 ± 0.03 ^b^	1.84 ± 0.04 ^cd^	1.84 ± 0.02 ^cd^
*ALT (u/L)*	12.34 ± 10.18 ^a^	76.08± 11.29 ^bc^	81.00± 26.08 ^cd^	78.11 ± 13.21 ^bd^
*AST (u/L)*	79.18 ± 5.39 ^a^	144.03± 12.01 ^b^	84.27± 4.19 ^c^	80.59 ± 6.13 ^ac^
*ALP (u/L)*	154.22 ± 7.07 ^a^	248.76 ± 9.26 ^b^	175.08 ± 6.15 ^cd^	172.31 ± 8.33 ^cd^
*Creatinine (µmol/L)*	2.19 ± 0.16 ^a^	8.00 ± 0.6 ^b^	5.61 ± 0.13 ^cd^	6.02 ± 0.67 ^cd^
*Urea (mg/dL)*	18.95 ± 3.11 ^a^	33.17 ± 1.94 ^b^	23.09 ± 2.13 ^c^	27.12 ± 3.15 ^d^
*Uric Acid (µmol/L)*	2.00 ± 0.08 ^a^	6.03 ± 1.33 ^b^	3.04 ± 0.99 ^cd^	3.11 ± 1.04 ^cd^
*LDH (u/L)*	607.19 ± 10.11 ^a^	854.47± 21.16 ^b^	677.16 ± 29.17 ^c^	644.82 ± 11.1 ^d^

Diet A (control 2.0 μg AFB_1_/kg + 3.0 mg FB_1_/kg); Diet B (19.7 μg AFB_1_/kg + 28.5 mg FB_1_/kg); Diet C (lanthanum chloride 200.0 mg/kg and 19.7 μg AFB_1_/kg + 28.5 mg FB_1_/kg); Diet D (lanthanum chloride 400.0 mg/kg and 19.7 μg AFB_1_/kg + 28.5 mg FB_1_/kg). Rows with different superscripts are significantly different (*p* < 0.05).

**Table 7:** Serum biochemical profile of juvenile *Clarias gariepinus* catfish fed diets amended with lanthanum chloride and mixtures of aflatoxin B1 and fumonisin B1 for 56 days.

*Parameter*	*Diet A (Control)*	*Diet B*	*Diet C*	*Diet D*
*Total Protein (g/dL)*	5.63 ± 0.55 ^a^	6.08 ± 0.23 ^abcd^	5.99 ± 0.28 ^bcd^	5.85 ± 0.34 ^abc^
*Albumin (g/dL)*	3.76 ± 0.03 ^a^	4.06 ± 0.12 ^be^	3.93 ± 0.06 ^c^	3.82 ± 0.02 ^d^
*Globulin (g/dL)*	1.87 ± 0.01 ^a^	2.02 ± 0.03 ^be^	2.06 ± 0.01 ^c^	2.03 ± 0.02 ^d^
*ALT (u/L)*	13.61 ± 0.31 ^a^	54.93 ± 2.17 ^b^	25.00 ± 1.10 ^c^	27.09 ± 1.15 ^d^
*AST (u/L)*	83.24 ± 1.88 ^a^	159.07 ± 9.01 ^b^	92.8 ± 2.19 ^cd^	94.01 ± 3.24 ^cd^
*ALP (u/L)*	162.01 ± 10.02 ^a^	243.91 ± 6.24 ^b^	183.2 ± 7.1 ^cd^	185.3 ± 6.5 ^cd^
*Creatinine (µmol/L)*	2.24 ± 0.19 ^a^	6.39 ± 0.01 ^b^	3.25 ± 0.17 ^cd^	3.15 ± 0.43 ^cd^
*Urea (mg/dL)*	20.38 ± 2.17 ^a^	31.68 ± 1.81 ^b^	26.39 ± 3.14 ^c^	24.28 ± 1.19 ^d^
*Uric Acid (µmol/L)*	1.83 ± 0.11 ^a^	5.07 ± 0.19 ^b^	2.91 ± 0.01 ^c^	3.06 ± 0.13 ^d^
*LDH (u/L)*	593.82 ± 12.19 ^a^	965.5 ± 34.28 ^b^	617.8 ± 19.0 ^ce^	637.9 ± 18.1 ^d^

Diet A (control 2.0 μg AFB_1_/kg + 3.0 mg FB_1_/kg); Diet B (19.7 μg AFB_1_/kg + 28.5 mg FB_1_/kg); Diet C (lanthanum chloride 200.0 mg/kg and 19.7 μg AFB_1_/kg + 28.5 mg FB_1_/kg); Diet D (lanthanum chloride 400.0 mg/kg and 19.7 μg AFB_1_/kg + 28.5 mg FB_1_/kg). Rows with different superscripts are significantly different (*p* < 0.05).

## Data Availability

The data presented in this study are available on request from the corresponding author.
